# Identification of patients at high risk for brain death using an automated digital screening tool: a prospective diagnostic accuracy study

**DOI:** 10.1007/s00415-023-11938-1

**Published:** 2023-08-25

**Authors:** Daniela Schoene, Norman Freigang, Anne Trabitzsch, Konrad Pleul, Daniel P. O. Kaiser, Martin Roessler, Simon Winzer, Christian Hugo, Albrecht Günther, Volker Puetz, Kristian Barlinn

**Affiliations:** 1 Department of Neurology, Faculty of Medicine and University Hospital Carl Gustav Carus, Technische Universität Dresden, Fetscherstraße 74, 01307 Dresden, Germany; 2 Department of Surgery, Faculty of Medicine and University Hospital Carl Gustav Carus, Technische Universität Dresden, Dresden, Germany; 3 Deutsche Stiftung Organtransplantation (DSO), Frankfurt am Main, Germany; 4 Institute of Neuroradiology, Faculty of Medicine and University Hospital Carl Gustav Carus, Technische Universität Dresden, Dresden, Germany; 5 BARMER Institute for Health Care System Research (Bifg), Berlin, Germany; 6 Division of Nephrology, Department of Internal Medicine III, Faculty of Medicine and University Hospital Carl Gustav Carus, Technische Universität Dresden, Dresden, Germany; 7 Department of Neurology, University Hospital Jena, Jena, Germany

**Keywords:** Death by neurologic criteria, Brain death, Organ donor identification, Diagnostic accuracy

## Abstract

**Background:**

An automated digital screening tool (DETECT) has been developed to aid in the early identification of patients who are at risk of developing brain death during critical care.

**Methods:**

This prospective diagnostic accuracy study included consecutive patients ≥ 18 years admitted to neurocritical care for primary or secondary acute brain injury. The DETECT screening tool searched routinely monitored patient data in the electronic medical records every 12 h for a combination of coma and absence of bilateral pupillary light reflexes. In parallel, daily neurological assessment was performed by expert neurointensivists in all patients blinded to the index test results. The primary target condition was the eventual diagnosis of brain death. Estimates of diagnostic accuracy along with their 95%-confidence intervals were calculated to assess the screening performance of DETECT.

**Results:**

During the 12-month study period, 414 patients underwent neurological assessment, with 8 (1.9%) confirmed cases of brain death. DETECT identified 54 positive patients and sent 281 notifications including 227 repeat notifications. The screening tool had a sensitivity of 100% (95% CI 63.1–100%) in identifying patients who eventually developed brain death, with no false negatives. The mean time from notification to confirmed diagnosis of brain death was 3.6 ± 3.2 days. Specificity was 88.7% (95% CI 85.2–91.6%), with 46 false positives. The overall accuracy of DETECT for confirmed brain death was 88.9% (95% CI 85.5–91.8%).

**Conclusions:**

Our findings suggest that an automated digital screening tool that utilizes routinely monitored clinical data may aid in the early identification of patients at risk of developing brain death.

## Introduction

Organ donation is crucial for patients with organ failure to improve quality of life and increase chances of survival [1]. However, the shortage of available organs has resulted in long waiting lists, leaving many patients without access to organ transplantation. In Europe alone, an estimated 20 patients per day die while waiting for an organ transplant [2]. In Germany, waiting lists for organ donation are particularly long compared to other countries with a recent organ donation rate of ten per one million inhabitants, reflecting the country’s consistently low organ donation rates for years [3, 4]. Deficiencies in identifying patients who may progress towards brain death after brain injury may have largely contributed to low organ donation rates in Germany, as indicated by a recent nationwide secondary analysis of 112 million hospitalized patients [5]. Despite a 13.9% increase in the number of potential organ donors (i.e., those who died in the presence of acute brain injury, were mechanically ventilated and had no medical contraindications for organ donation) between 2010 and 2015, there was a concurrent 18.7% decline in referrals to the German organ procurement organization (OPO) and a 32.3% decrease in the actual number of deceased organ donations. Furthermore, findings from an analysis of 7889 deceased patients with acute brain injuries raised similar concerns regarding the proper identification of potential organ donors in procurement hospitals [6]. Notably, 73 patients who were retrospectively classified as potentially at risk of developing brain death were ultimately not subjected to a corresponding neurological evaluation.

To address these shortcomings in potential donor identification, an automated digital screening tool (DETECT; screening for potential brain DEath in paTiEnts with severe brain damage and clinically asCerTained loss of cerebral functions) was developed to prospectively identify intensive care patients who are at risk of developing brain death [7, 8]. In an analysis of 309 deceased patients with severe brain injury, the pilot implementation of DETECT in intensive care units of a tertiary care hospital was associated with a 93% decrease in the risk of missing patients who were retrospectively classified as impending brain death and thus being potential donors, compared to a period before implementation.

In view of aforementioned considerations, we conducted a diagnostic accuracy study to prospectively determine the screening performance of DETECT in identifying neurocritically ill patients with impending brain death. We hypothesized that DETECT could provide reliable information that would increase clinicians’ awareness of these patients and facilitate their early referral to neurointensivists for further evaluation.

## Methods

### Study design and patient population

**Fig. 1:**
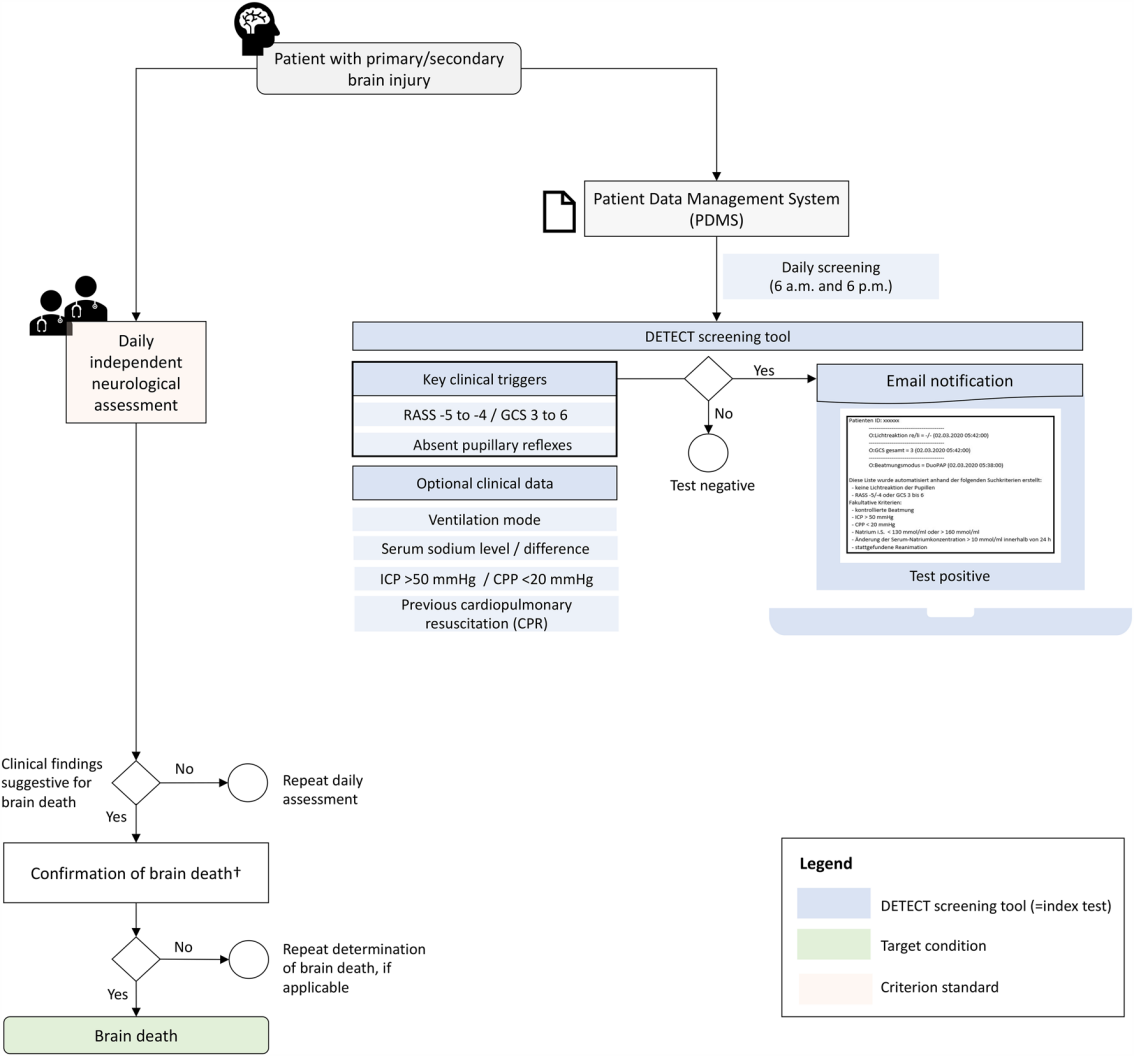
Study design. ^†^Determination according to the guideline of the German Medical Association [14]. *RASS* Richmond Agitation Sedation Scale, *GCS* Glasgow Coma Scale, *ICP* intracranial pressure, *CPP* cerebral perfusion pressure, *F/U* follow up

This was a prospective validation study that adopted the Standards for Reporting of Diagnostic Accuracy (STARD) guidelines [9]. We enrolled consecutive patients aged 18 years or older who were admitted between February 2020 and January 2021 to the 12-bed neurocritical care unit of a tertiary care hospital in Germany. This study included all patients with acute neurological diseases primarily or secondary affecting the brain. Patients with peripheral neurological disorders such as myasthenia gravis or Guillain-Barré-syndrome and those admitted to the neurocritical care unit for non-neurological reasons were excluded. The DETECT screening tool was considered the index test to identify patients exhibiting clinical findings indicative of impending brain death. The primary target condition in our study was the final diagnosis of brain death, as determined by board-certified neurologists with subspecialty training in neurocritical care. In adherence to the study protocol, we prospectively collected data on demographics, clinical information, treatment specifics including analgosedation regimens, laboratory results, neuroimaging findings, and the actual frequency of deceased organ donation. An illustration of the study design is provided in Fig. 1.

### Automated digital screening—DETECT

Our collaborative group developed an automated search algorithm to screen routinely monitored patient data in the electronic medical records system (ICM^®^, Dräger Medical, Lübeck) of adult intensive care units at the University Hospital Dresden [7, 8]. Meanwhile, the technical compatibility of DETECT has been extended to encompass the majority of widely used electronic medical records systems in intensive care. Detailed information about its programming code and technical integration with electronic medical records systems can be found elsewhere [10]. In brief, DETECT cyclically processes incoming data from the corresponding electronic medical record system and generates notifications via the hospital’s internal email server to transplant coordinators or a custom-configured group. Diverse interfaces (such as XML, REST, HL7v2, or FHIR) can be employed based on the specific electronic medical record system used, enabling the capture of key clinical parameters necessary for the predefined screening algorithm.

The screening targeted a combination of coma, indicated by a Richmond Agitation Sedation Scale (RASS) score of − 4 or − 5 or Glasgow Coma Scale (GCS) score of 6–3, along with manually assessed absence of bilateral pupillary light reflexes, both of which are considered early indicators of impending brain death [11]. Following critical care guideline recommendations, the RASS or GCS scores and pupillary function are routinely monitored by critical care staff and documented in the electronic medical records [12, 13]. The search algorithm ran every 12 h, and upon a positive result, an automated notification was dispatched via the hospital’s email server to the corresponding transplant coordinators and intensivists. The notification included demographic data of the patient, date and time of the registered condition, and optional clinical and laboratory details. This optional information included whether a patient was on mechanical ventilation without spontaneous breathing, had intracranial pressure (if monitored) above 50 mmHg and/or cerebral perfusion pressure below 20 mmHg, and had serum sodium levels above 160 mmol/L or changes greater than 10 mmol/L in the past 24 h, all of which may point towards an irreversible loss of brain function. The notification also contained information on whether the patient had undergone cardiopulmonary resuscitation prior to or during hospitalization. The email sent to the transplant coordinators aimed to prompt clinical assessment of the reported patient and, if necessary, initiate a guideline-based brain death examination in collaboration with the treating neurointensivist.

### Serial neurological assessment

Two expert neurointensivists conducted daily neurological assessment on all study participants during their ICU stay to establish the clinical reference standard for identifying patients at risk of impending brain death blinded to the results of the index test. Neurological assessment and the index test were performed within 24 h from each other. The study protocol encompassed an assessment of the level of consciousness, brainstem reflexes (including pupillary, corneal, gag and vestibulo-ocular reflexes) and limb motor functions. The study physicians had access to all clinical, neuroimaging, and laboratory information gathered during the patients’ hospital stay. Patients with clinically suspected impending brain death (such as those with severe brain damage, persistent coma, and absence of brain stem reflexes) subsequently underwent a standardized clinical examination to confirm or exclude brain death in accordance with the guidelines of the German Medical Association [14]. Ancillary diagnostic tests, including transcranial ultrasonography, computed tomography angiography, and electroencephalography, were used for brain death determination as needed. If brain death could not be confirmed, a repeat examination was performed afterwards at the discretion of the treating neurointensivists.

### Statistical analysis

Normally distributed continuous variables are presented as mean ± standard deviation (SD), while skewed distributed data are presented as median (interquartile range, IQR). Categorical variables are presented as percentages. To assess the performance of the DETECT screening tool, we calculated its sensitivity, specificity, positive predictive value (PPV), negative predictive value (NPV), and overall accuracy. The primary outcome was the identification of patients who eventually developed brain death during their hospitalization. True-positive, false-positive, true-negative, and false-negative values were computed for this purpose. The corresponding 95% Wilson confidence intervals (95% CI) were computed to assess the precision of the accuracy estimates. Differences in the distribution of variables between subgroups of interest were assessed using appropriate statistical tests, including Wilcoxon rank sum test, Student’s *t* test, Chi-squared test, or Fisher’s Exact test. Bivariate logistic regression was used to examine associations between clinical variables and DETECT screening results. The significance level was set at 0.05. Statistical analyses were conducted using STATA (version 16.1, StataCorp, College Station, TX) and MedCalc (version 19.8, MedCalc Ltd., Ostend, Belgium) softwares.

## Results

### Study population

**Fig. 2:**
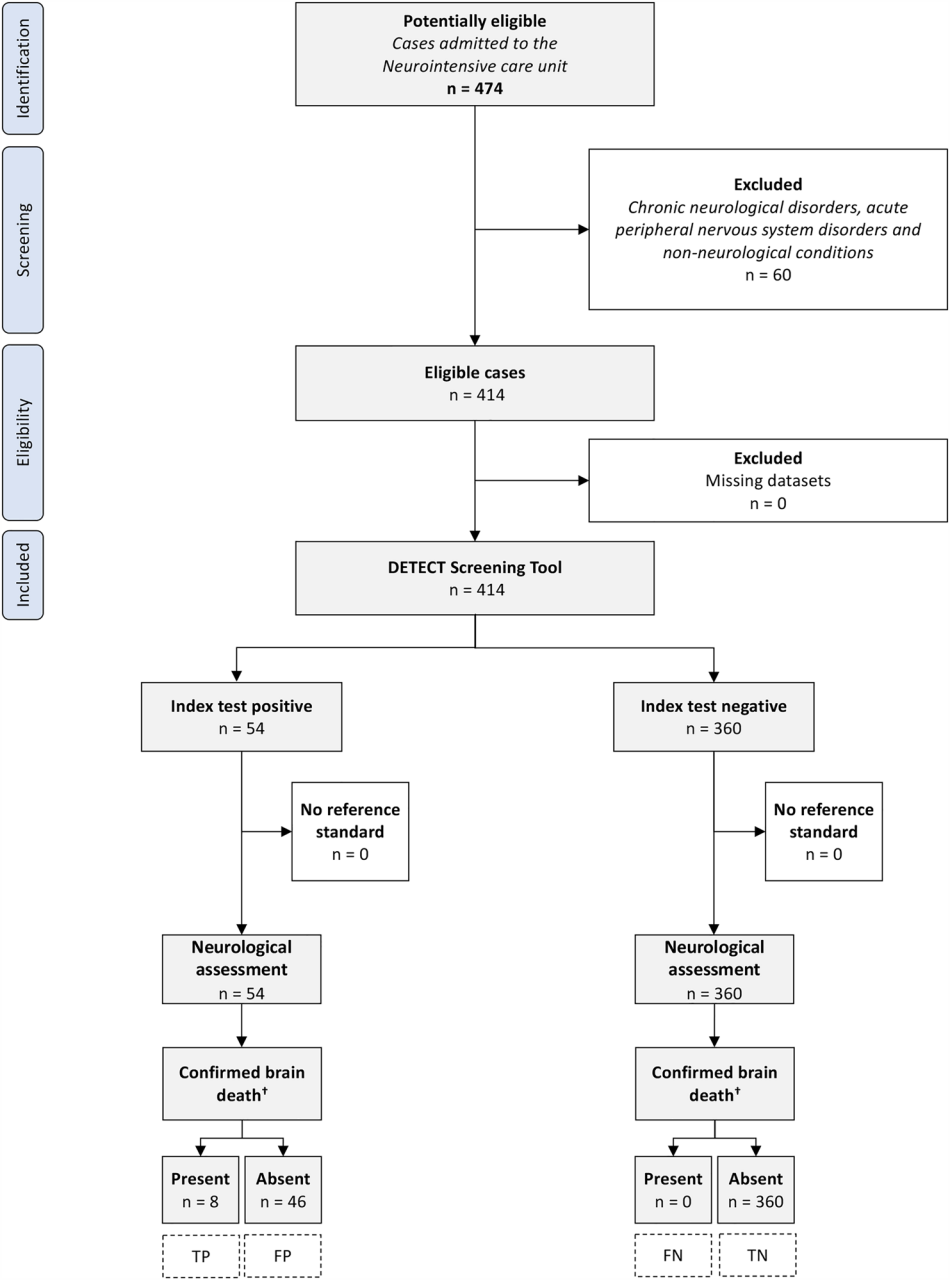
Flow chart of the study participants. ^†^According to the guideline of the German Medical Association [14]. *TP* true positive, *TN* true negative, *FP* false positive, *FN* false negative

During the 12-months study period, 414 (87.3%) of 474 patient admissions to the neurocritical care unit met the inclusion criteria for this diagnostic accuracy study. The flow chart of the study patients including diagnostic outcomes is shown in Fig. 2. The mean age of the study population was 70.8 ± 14.8 years, 45.9% were female, the median baseline GCS score was 11 (IQR 5–14) and the mean length of ICU stay was 9.1 ± 8.6 days. The most common reasons for admission to the neurocritical care unit were acute ischemic stroke (290/414 [70.1%]), spontaneous intracerebral hemorrhage (53/414 [12.8%]), seizure or status epilepticus (34/414 [8.2%]) and meningoencephalitis (13/414 [3.1%]). A total of 174 patients (42%) required controlled mechanical ventilation during their ICU stay. Among patients with acute ischemic or hemorrhagic stroke, the median baseline National Institutes of Health Stroke Scale (NIHSS) score was 16 (IQR 8–24) points. Table 1 provides further details on the clinical characteristics of the study population. No missing data were present for any baseline or outcome variables of interest.

**Table 1:** Characteristics of brain injuries among the study population (n = 414)

Primary brain injury, *n* (%)	
Acute ischemic stroke	290 (70.1)
Malignant middle cerebral artery infarction	41 (9.9)
Space-occupying cerebellar infarction	7 (1.7)
Secondary symptomatic intracerebral bleeding	27 (6.5)
Spontaneous intracerebral hemorrhage	53 (12.8)
Intraventricular extension	38 (9.2)
Hematoma expansion within 72 h	9 (2.2)
Seizure / status epilepticus	34 (8.2)
Acute meningoencephalitis	13 (3.1)
Bacterial	4 (1.0)
Viral	7 (1.7)
Subarachnoid hemorrhage	4 (1.0)
Subdural hematoma	4 (1.0)
Traumatic brain injury	2 (0.5)
Posterior reversible encephalopathy syndrome	3 (0.7)
Cerebral venous sinus thrombosis	1 (0.2)
Other (Brain metastases, Creutzfeldt–Jakob disease, Spontaneous intracranial hypotension, Neurosarcoidosis)	4 (1.0)
Secondary brain injury, *n* (%)	
Hypoxic ischemic encephalopathy	6 (1.5)
Intracranial cerebral pressure complications, *n* (%)	
Brain herniation	24 (5.8)
Cerebrospinal fluid circulatory dysfunction	38 (9.2)
Therapeutic interventions, *n* (%)	
Decompressive hemicraniectomy	26 (6.3)
Atlantooccipital trepanation	4 (1.0)
External ventricular drainage	28 (6.8)
Hyperosmolar therapy	14 (3.4)

### Distribution of the target condition

**Fig. 3:**
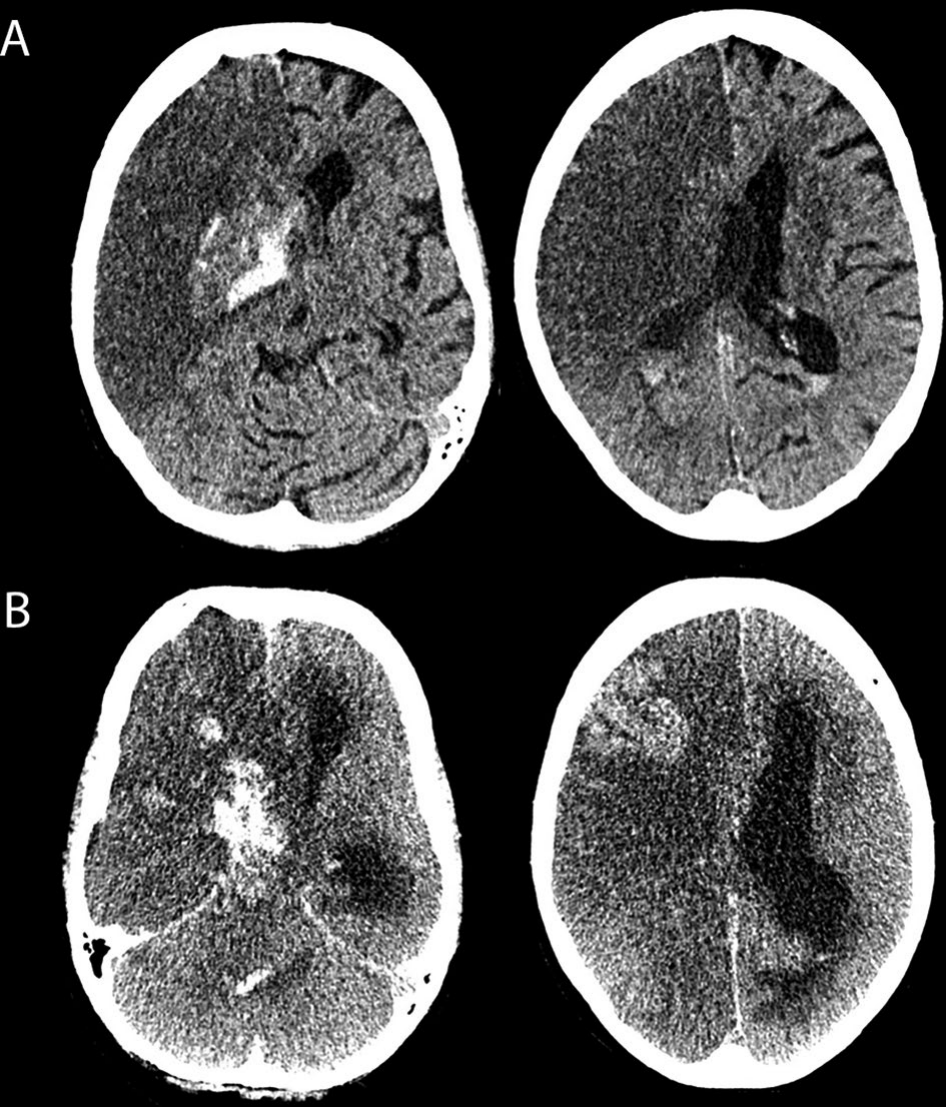
Patient with large right-sided hemispheric infarction that resulted in cerebral edema, secondary intracerebral hemorrhage, intraventricular hemorrhage, hydrocephalus and transtentorial herniation. The patient was screened positive by DETECT on day 4 (6 a.m.) following admission to neurocritical care. **A** Early follow-up CT on day 2 used for independent neuroimaging assessment for the potential of developing brain death. **B** CT scan on day 5 when brain death was confirmed

Out of 414 patients who underwent prospective neurological assessment, 8 (1.9%) were eventually confirmed to have brain death through standardized clinical determination. Among them, five patients had space-occupying large hemispheric infarction, one had space-occupying cerebellar infarction, one had intracerebral hemorrhage, and one had bacterial meningoencephalitis complicated by septic cerebral venous thrombosis. Seven of these patients underwent deceased organ donation (Fig. 3).

### Screening test performance

Overall, the DETECT screening tool identified 54 patients with positive test results and dispatched a total of 281 notifications, including 227 repeated notifications. The mean time between the index event and the initial notification was 3.2 ± 4.8 days (median 1.4, IQR 0.7–3.5). Of these 54 patients, 34 were repeatedly detected with a median of 4.5 (IQR 2–10) notifications per patient over a mean duration of 4 ± 3.2 days (median 2.5, IQR 1.5–6.5). Repeated notifications were dispatched every 14.5 ± 10 h (median 12, IQR 11.9–12.1).

As per notifications and verified by the study physicians, 90.7% (49/54) of the positively screened patients were on controlled mechanical ventilation, 11.1% (6/54) had intracranial pressure exceeding 50 mmHg and 7.4% (4/54) had cerebral perfusion pressure below 20 mmHg at least once during their screening period. Additionally, 13% (7/54) had serum sodium levels above 160 mmol/L and 46.3% (25/54) showed changes greater than 10 mmol/l in the past 24 h. Cardiopulmonary resuscitation was performed on 11.1% (6/54) of the patients.

The DETECT screening tool accurately identified all confirmed cases of brain death with a sensitivity of 100% (95% CI 63.1–100%), indicating no false negatives. The mean time between initial notification and final diagnosis of brain death was 3.6 ± 3.2 days (median 2.9, IQR 1.6–4.8). Seven out of the eight (87.5%) confirmed brain dead patients were repeatedly detected with a median of 7 (IQR 3.5–11.5) notifications per patient, while the remaining patient was detected once. The specificity of DETECT was 88.7% (95% CI 85.2–91.6%) corresponding to 46 false positive cases. In comparison to patients accurately classified as negative by the screening tool, false positives demonstrated a lower baseline GCS score (median 6, IQR 3-12 vs. median 11, IQR 6-14; *p* = 0.001), higher rates of any vertebrobasilar stroke (30.4% vs. 13.1%; *p* = 0.002) and space-occupying cerebellar stroke (8.7% vs. 0.6%; *p* = 0.002), a greater necessity for mechanical ventilation (87% vs. 35%; *p* < 0.001), a more frequent use of analgosedation throughout the ICU stay (89.1% vs. 56.7%; *p* < 0.001) and an increased frequency of intracranial pressure complications including brain herniation (10.9% vs. 3.1%; *p* = 0.025) and cerebrospinal fluid circulatory dysfunction (26.1% vs. 5.3%; *p* < 0.001). No significant differences were identified with regard to other causes of primary or secondary brain injury, demographic characteristics, or relevant clinical data. Notably, analgosedation emerged as a predictor of positive screening results in the entire study cohort (OR 6.1, 95% CI 2.6–14.7; *p* < 0.001). When restricting the analysis to the 34 cases with repeated notifications, the specificity increased to 93.4% (95% CI 90.5–95.6%).

Within the subgroup of 174 patients who required mechanical ventilation during their ICU stay, the sensitivity of the DETECT screening tool for identifying those who eventually progressed to brain death remained unchanged. However, the specificity decreased to 75.9% (95% CI 68.7–82.2%), which could be attributed to the prevalent utilization of analgosedation (94.3%) in these patients. Additionally, any vertebrobasilar stroke (35% vs. 15.9%), space-occupying cerebellar stroke (10% vs. 1.6%) and cerebrospinal fluid circulatory complications (27.5% vs. 10.3%) were more frequently observed among falsely identified cases compared to true negatives (*p* < 0.05).

The overall accuracy of DETECT for confirmed brain death was 88.9% (95% CI 85.5–91.8%). Further accuracy estimates are detailed in Table 2.

**Table 2:** DETECT screening test performance

Accuracy estimates, % (95% CI)	Confirmed brain death^†^
Sensitivity	100 (63.1–100)
Specificity	88.7 (85.2–91.6)
Positive predictive value	14.8 (11.7–18.6)
Negative predictive value	100
Overall accuracy	88.9 (85.5–91.8)

*CI* confidence interval

^†^Determination according to the guideline of the German Medical Association [14]

### Discharge outcomes of positively screened patients

Among the 54 patients identified as positive by DETECT, 30 patients (55.6%) died during neurocritical care, with 7 (12.9%) of them undergoing deceased organ donation. Twelve patients (22.2%) were discharged for acute rehabilitation, one patient (1.9%) was referred to the anesthetic intensive care unit and 11 patients (20.4%) were directed to non-intensive care in-hospital units.

## Discussion

The findings of this diagnostic accuracy study demonstrate the ability of the DETECT screening tool to accurately identify patients with acute brain injury who are at high risk of progressing to brain death during neurocritical care. Notably, the tool did not miss patients who were eventually confirmed as brain dead, and the false detection rate among negatively confirmed patients was only one in ten. These results highlight the potential effectiveness of automated screening for key clinical triggers in identifying potential organ donors.

Timely identification and referral of potential organ donors is widely recognized as a key strategy for addressing the global shortage of life-saving transplantable organs [15–17]. Digital support systems utilizing patient health records have been shown to enhance early recognition and clinical decision-making for various conditions, including sepsis, venous thromboembolism, acute kidney injury, and pediatric appendicitis [18–21]. In a recent retrospective study of adult patients, electronically automated referrals to an OPO based on clinical triggers such as mechanical ventilation, acute neurocritical injury and a GCS score of ≤ 5 was associated with a 45% increase in monthly donor referrals and a 92% increase in monthly deceased donors compared to a pre-implementation period when referrals were made manually by the ICU healthcare team [22]. Another retrospective study evaluated the effects of an automated clinical decision support system that sent an email notification to the OPO when clinical findings suggestive of impending brain death were documented for pediatric patients [23]. The system was triggered by the presence of bilateral unresponsive pupils, mechanical ventilation and either an absent gag reflex or a GCS score of ≤ 5 within the previous 24 h in the electronic nursing assessments. The implementation of the system resulted in a shorter time to OPO notification and a higher rate of conversion from potential to actual donors compared to the period before implementation. However, there was no difference in the frequency of actual brain death determination between the two periods.

While these studies suggest that automated potential donor identification systems can be effective, their conclusions are limited by the lack of a parallel reference standard, such as neurological assessment and the possibility of external factors influencing the potential donor pool. Additionally, the donor referral technology used in the former study relied solely on clinical data available at the time of ICU admission [22]. In contrast, the findings of the present study suggest that repeat alerts during ICU stay may further improve the diagnostic accuracy of automated identification of potential donors. In fact, nearly 90% of eventual brain-dead patients in this study were repeatedly identified, with a median of seven notifications per patient and up to 10 days prior to brain death determination. Therefore, repeat detection of neurologic key symptoms may indicate genuine evolution of critical intracranial pressure due to devastating brain injury, rather than being attributed to anesthetic effects such as suppression of brainstem reflexes [24].

Variations in healthcare practices across different countries hinder the comparability of potential donor identification approaches [24]. In the United States, the standard procedure is to directly notify the OPO upon admission of a patient meeting clinical criteria suggestive of impending brain death to the ICU. In contrast, the DETECT screening tool was developed to align with the prevailing practice in Germany and other European countries, where the hospital’s transplant coordination team is informed about a potential donor and establishes contact with the OPO after confirming brain death and approaching the family. Notifying the hospital’s transplant coordinators instead of the OPO in patients suggestive of impending brain death may have various benefits, including a closer evaluation of potential donors and their families by the transplant coordinators in collaboration with the treating intensivists and reduced burden on the OPO with fewer futile notifications [25].

The average time between initial automated notification to the transplant team and determination of brain death was 3.6 days in our study, allowing for sufficient time to conduct comprehensive neurological assessments, communicate with the family, and obtain necessary authorizations while ensuring timely determination of brain death. Prolonging brain death determination may jeopardize the overall function of viable organs and their procurement, as brain death can initiate severe systemic inflammation with cytokine storms similar to those seen in sepsis, hemodynamic instability, and unfavorable hormonal changes [26–28].

The automated screening algorithm implemented by DETECT employs simple yet specific neurologic criteria [11]. In contrast to other electronic support systems, mechanical ventilation was not mandated as a criterion for screening positivity, thus potentially expanding the pool of eligible donors beyond those who are mechanically ventilated. Although still uncommon in Germany, early identification and referral of possible organ donors from the emergency department to elective non-therapeutic intensive care is thought to be a contributing factor to Spain’s organ donation rate of 40 donors per one million, positioning it as the global leader in organ donation [17, 29]. The lower specificity of DETECT observed in mechanically ventilated patients may be attributed to the concurrent use of analgosedatives, which were administered to over 90% of these patients. Notably, patients receiving analgosedatives were six times more likely to trigger a positive result from the screening tool. Moreover, analysis of the entire study cohort unveiled distinct characteristics that were more frequently observed in falsely detected cases than in true negatives—any vertebrobasilar stroke, intracranial pressure-related complications, mechanical ventilation and analgosedation usage—conditions that could potentially explain compromised pupillary light reflexes and impaired vigilance, without necessarily indicating progressing to brain death. However, it is important to note that false positive screening for potential donors is considered less concerning than false negative screening. The former does not harm those who are mistakenly identified, whereas the latter can result in failure to identify potential donors and a missed opportunity to prevent death or disability in transplant candidates. While relying solely on routinely documented clinical assessments for automated screening saves ICU personnel resources, the potential enhancement of specificity through the integration of additional clinical data from bispectral index monitoring, intracranial pressure monitoring, biomarker studies, or machine learning techniques warrants further investigation [30–33].

This study has several strengths. Firstly, it is the first to prospectively validate an automated digital screening approach for identifying key clinical triggers of impending brain death, using simultaneous and blinded neurological assessment as the reference standard. Unlike prior research on automated donor identification technology, this diagnostic accuracy study builds on a pilot exploration of DETECT at a tertiary care hospital. Our previous pilot study among 114 ICU beds revealed that DETECT was associated with a 93% lower risk of failing to identify potential brain-dead donors since its implementation in 2018, which prompted a subsequent investigation into its internal validity [7]. In the current study, we therefore focused on a neurocritical care cohort with an expected higher incidence of brain death, a rare outcome, which adds to the significance of our findings. Yet this approach may have limited the generalizability of our findings to non-neurological ICU cohorts. The low positive predictive value we observed could be due to the inherent rarity of brain death defined as the target condition in this study. Nonetheless, implementing DETECT in non-neurological ICUs that naturally lack neurocritical care expertise could be particularly beneficial in terms of potential donor identification.

Limitations of this study include the monocentric design, and the fact that we did not investigate the extent to which healthcare providers effectively utilized the information conveyed by the automated notifications or whether repeated notifications contributed to alarm fatigue, as these aspects were beyond the scope of this diagnostic accuracy study. However, it is crucial to consider the potential implications of alarm fatigue in real-world clinical practice when using DETECT. An increased number of notifications could desensitize transplant coordination teams and intensivists, leading to missed responses to critical information on potential organ donors. Moreover, the subsequent steps following potential donor identification are largely reliant on the initiative of the transplant coordination team and treating intensivists, potentially affecting the actual donation rate achieved by the DETECT screening tool. An upcoming multicenter randomized controlled trial on the efficacy of DETECT will therefore gain insights into how healthcare providers receive and manage notifications. Furthermore, future enhancements to DETECT aim to alleviate alarm fatigue and enhance its clinical utility, including customization options enabling selected healthcare professionals to tailor daily notifications—such as excluding patients under deep sedation for medical reasons or those with pre-existing tumors ineligible for organ donation—from the daily screening process. Additionally, our study might have been susceptible to bias arising from competing risks, which may have impacted the frequency of brain death in our study population due to the occurrence of death from other causes prior to progression to brain death (e.g. withdrawal of life-sustaining therapies for perceived poor neurologic prognosis). As a result, this bias could have resulted in an underestimation of the screening tool’s sensitivity, as potential brain death cases might have been excluded, and an overestimation of specificity, by considering potential brain death cases as true negatives. Lastly, DETECT was initially designed to be compatible with the electronic medical records systems employed in the study center restricting its external validity. However, its technical compatibility has been expended to ensure cost-neutral compliance with the majority of widely used electronic medical records systems in intensive care [10].

## Conclusions

Our findings suggest that implementing an automated digital screening tool to monitor routine clinical data can facilitate the early detection of patients who may be at risk of developing brain death during neurocritical care. To assess the efficacy of DETECT in improving potential donor identification and increasing organ donation rates, a multicenter randomized controlled trial is currently underway.

## Data Availability

The datasets used and analyzed during the current study are available from the corresponding author on reasonable request.

## References

[1] Vanholder R, Domínguez-Gil B, Busic M (2021). Organ donation and transplantation: a multi-stakeholder call to action. Nat Rev Nephrol.

[2] European Day for Organ Donation and Transplantation (EODD) - European Directorate for the Quality of Medicines & HealthCare. https://www.edqm.eu/en/eodd. Accessed 15 May 2023

[3] Lewis A, Koukoura A, Tsianos GI (2021). Organ donation in the US and Europe: the supply vs demand imbalance. Transplant Rev (Orlando).

[4] Organspende: Ruf nach erneuter Reform. https://www.aerzteblatt.de/archiv/229477/Organspende-Ruf-nach-erneuter-Reform. Accessed 6 Jul 2023

[5] Schulte K, Kunzendorf U, Feldkamp T (2018). Decline in organ donation in Germany: a nationwide secondary analysis of all inpatient cases. Dtsch Arztebl Int.

[6] Brauer M, Günther A, Pleul K (2019). How many potential organ donors are there really?: Retrospective analysis of why determination of irreversible loss of brain function was not performed in deceased patients with relevant brain damage. Anaesthesist.

[7] Trabitzsch A, Pleul K, Barlinn K (2021). Automatisiertes elektronisches Screeningtool (DETECT) zur Erkennung des potenziell irreversiblen Hirnfunktionsausfalls. Dtsch Arztebl Int.

[8] Klick R (2022). DETECT should force us to think about the situation of our ICUs. Dtsch Arztebl Int.

[9] Cohen JF, Korevaar DA, Altman DG (2016). STARD 2015 guidelines for reporting diagnostic accuracy studies: explanation and elaboration. BMJ Open.

[10] pub / detect · GitLab. https://gitlab.ukdd.de/pub/detect. Accessed 6 Jul 2023

[11] Squires JE, Coughlin M, Dorrance K (2018). Criteria to identify a potential deceased organ donor: a systematic review. Crit Care Med.

[12] Devlin JW, Skrobik Y, Gélinas C (2018). Clinical practice guidelines for the prevention and management of pain, agitation/sedation, delirium, immobility, and sleep disruption in adult patients in the ICU. Crit Care Med.

[13] Kotloff RM, Blosser S, Fulda GJ (2015). Management of the potential organ donor in the ICU: Society of Critical Care Medicine/American College of Chest Physicians/Association of Organ Procurement Organizations Consensus Statement. Crit Care Med.

[14] Richtlinie gemäß § 16 Abs. 1 S. 1 Nr. 1 TPG für die Regeln zur Feststellung des Todes nach § 3 Abs. 1 S. 1 Nr. 2 TPG und die Verfahrensregeln zur Feststellung des endgültigen, nicht behebbaren Ausfalls der Gesamtfunktion des Großhirns, des Kleinhirns und des Hirnstamms nach § 3 Abs. 2 Nr. 2 TPG, Fünfte Fortschreibung Vorwort. 10.3238/arztebl.2022.rl_hirnfunktionsausfall_02

[15] Zavalkoff S, Shemie SD, Grimshaw JM (2019). Potential organ donor identification and system accountability: expert guidance from a Canadian consensus conference. Can J Anaesth.

[16] NICE (2011). Organ donation for transplantation: improving donor identification and consent rates for deceased organ donation. NHS Nice Clin Guideline.

[17] Matesanz R, Domínguez-Gil B, Coll E (2017). How Spain reached 40 deceased organ donors per million population. Am J Transplant.

[18] Colpaert K, Hoste EA, Steurbaut K (2012). Impact of real-time electronic alerting of acute kidney injury on therapeutic intervention and progression of RIFLE class. Crit Care Med.

[19] Amland RC, Lyons JJ, Greene TL, Haley JM (2015). A two-stage clinical decision support system for early recognition and stratification of patients with sepsis: an observational cohort study. JRSM Open.

[20] Amland RC, Dean BB, Yu HT (2015). Computerized clinical decision support to prevent venous thromboembolism among hospitalized patients: proximal outcomes from a multiyear quality improvement project. J Healthc Qual.

[21] Kharbanda AB, Madhok M, Krause E (2016). Implementation of electronic clinical decision support for pediatric appendicitis. Pediatrics.

[22] Levan ML, Trahan C, Klitenic SB (2022). Short report: evaluating the effects of automated donor referral technology on deceased donor referrals. Transplant Direct.

[23] Zier JL, Spaulding AB, Finch M (2017). Improved time to notification of impending brain death and increased organ donation using an electronic clinical decision support system. Am J Transplant.

[24] Lewis A, Kirschen MP, Badenes R (2023). Quality improvement in the determination of death by neurologic criteria around the world. Crit Care.

[25] Martin-Loeches I, Sandiumenge A, Charpentier J (2019). Management of donation after brain death (DBD) in the ICU: the potential donor is identified, what’s next?. Intensive Care Med.

[26] Silva A, Arora S, Dhanani S (2022). Quality improvement tools to manage deceased organ donation processes: a scoping review protocol. Nurse Educ Pract.

[27] Tanim Anwar ASM, Lee JM (2019). Medical management of brain-dead organ donors. Acute and Critical Care.

[28] Schwarz P, Custódio G, Rheinheimer J (2018). Brain death-induced inflammatory activity is similar to sepsis-induced cytokine release. Cell Transplant.

[29] Akkas M, Demir MC (2019). Barriers to brain death notifications from emergency departments. Transplant Proc.

[30] Jouffroy R, Lamhaut L, Guyard A (2017). Early detection of brain death using the Bispectral Index (BIS) in patients treated by extracorporeal cardiopulmonary resuscitation (E-CPR) for refractory cardiac arrest. Resuscitation.

[31] Lopes Ideta MM, Oliveira LM, Gonçalves DB (2023). Qualitative evaluation of intracranial pressure slopes in patients undergoing brain death protocol. Brain Sci.

[32] Mozaffari K, Dejam D, Duong C (2021). Systematic review of serum biomarkers in traumatic brain injury. Cureus.

[33] Schweingruber N, Mader MMD, Wiehe A (2022). A recurrent machine learning model predicts intracranial hypertension in neurointensive care patients. Brain.

